# Identification of cerebral perfusion using arterial spin labeling in patients with seizures in acute settings

**DOI:** 10.1371/journal.pone.0173538

**Published:** 2017-03-14

**Authors:** Roh-Eul Yoo, Tae Jin Yun, Byung-Woo Yoon, Sang Kun Lee, Soon-Tae Lee, Koung Mi Kang, Seung Hong Choi, Ji-hoon Kim, Chul-Ho Sohn, Sun-Won Park, Moon Hee Han

**Affiliations:** 1 Institute of Radiation Medicine, Seoul National University Medical Research Center, Seoul, Republic of Korea; 2 Department of Radiology, Seoul National University Hospital, Seoul, Republic of Korea; 3 Department of Neurology, Seoul National University Hospital, Seoul, Republic of Korea; 4 Department of Radiology, Seoul National University Boramae Medical Center, Seoul, Republic of Korea; Henry Ford Health System, UNITED STATES

## Abstract

This study aimed to explore the utility of arterial spin labeling perfusion-weighted imaging (ASL-PWI) in patients with suspected seizures in acute settings. A total of 164 patients who underwent ASL-PWI for suspected seizures in acute settings (with final diagnoses of seizure [n = 129], poststroke seizure [n = 18], and seizure mimickers [n = 17]), were included in this retrospective study. Perfusion abnormality was analyzed for: (1) pattern, (2) multifocality, and (3) atypical distribution against vascular territories. Perfusion abnormality was detected in 39% (50/129) of the seizure patients, most (94%, 47/50) being the hyperperfusion pattern. Of the patients with perfusion abnormality, multifocality or hemispheric involvement and atypical distribution against vascular territory were revealed in 46% (23/50) and 98% (49/50), respectively. In addition, seizures showed characteristic features including hyperperfusion (with or without non-territorial distribution) on ASL-PWI, thus differentiating them from poststroke seizures or seizure mimickers. In patients in whom seizure focus could be localized on both EEG and ASL-PWI, the concordance rate was 77%. The present study demonstrates that ASL-PWI can provide information regarding cerebral perfusion status in patients with seizures in acute settings and has the potential to be used as a non-invasive imaging tool to identify the cerebral perfusion in patients with seizures.

## Introduction

A seizure increases metabolic demand in involved cerebral parenchyma, which is in turn accompanied by temporarily increased regional brain perfusion [1–3]. Therefore, analyzing cerebral perfusion and metabolic status has become a widely accepted method for evaluation of suspected seizures [2, 4].

As for the imaging evaluation in seizure patients, nuclear medicine perfusion techniques including positron emission tomography (PET) and single-photon emission CT (SPECT) have revealed increased cerebral perfusion in the critical period and decreased cerebral perfusion in the post-ictal period [5]. However, these techniques require separate evaluations over multiple days and radioactive materials must be prepared in advance. Therefore, the clinical utility of these techniques may be limited in acute settings. On the other hand, MRI is routinely used to rule out the possibility of underlying structural abnormalities in seizure patients [6]. According to previous reports, abnormal diffusion restriction on diffusion weighted image (DWI) can be found in patients with seizures [7]. Some investigators have used dynamic susceptibility contrast perfusion-weighted imaging (DSC-PWI) for patients with clinical seizure activities and have demonstrated that cerebral blood volume or cerebral blood flow (CBF) from DSC-PWI could also reflect the transient perfusion abnormality in seizures [8–13]. However, the validity of DWI and DSC-PWI for evaluating seizure patients has not been established.

Recently, arterial spin labeling perfusion-weighted imaging (ASL-PWI) has received increasing attention as a potential alternative to DSC-PWI. ASL-PWI, unlike DSC-PWI, is a completely noninvasive perfusion MR technique that uses magnetically labeled water as a diffusible tracer to measure cerebral blood flow values in a manner analogous to that used for PET or SPECT [14, 15]. Previous studies regarding the utility of ASL-PWI in seizure evaluation have shown promising results that ASL-PWI may delineate the time-related perfusion change in seizures and correlate well with DSC-PWI [10, 16–27]. However, the study populations in the previous studies are small and the assessment focusing on the acute setting has not been elucidated.

Therefore, the purpose of the present study was to explore the utility ASL-PWI for identifying the cerebral perfusion status in patients with suspected seizures in acute settings.

## Materials and methods

This retrospective study was approved by institutional review board of Seoul National University Hospital, and informed consent was waived.

### Patient selection

Database search of electronic medical charts from August 2012 to April 2015 identified 194 patients who underwent MR imaging, including DWI and ASL-PWI for suspected seizures in acute settings (i.e., seizures in the emergency room or seizures in hospitalized patients). The final diagnoses of seizure were determined by neurologists based on seizure semiology, electroencephalogram (EEG) findings, imaging modalities (including conventional MR sequences, DWI, MR angiography, and ictal SPECT). Thirty patients were excluded for the following reasons: 1) uncertain diagnosis (n = 12), and 2) poor ASL image quality due to inadequate acquisition times or artifacts (n = 18). As a result, 164 patients were included in this study.

Final diagnoses of the patients were classified into the following three groups: 1) seizure, 2) poststroke seizure, and 3) seizure mimickers. For seizures, time intervals between the last seizure events and MR scans were recorded and categorized as follows: 1) the time interval equal to or shorter than 5 hours, and 2) the time interval longer than 5 hours [10]. Seizures were further analyzed for the type of presenting symptoms as follows: 1) convulsive if at least one event of convulsive movement occurred, and 2) nonconvulsive if not. In terms of the seizure semiology, seizures were further classified into generalized seizures and partial seizures. In addition, poststroke seizures were further divided into the following three subgroups according to their time of onset: 1) onset seizures which occurred within 24 hours of stroke onset, 2) early seizures which occurred within 1 week after stroke, and 3) late seizures which occurred thereafter [28].

### MR imaging protocol

All MR images were acquired at a 1.5T (Signa HDxt, GE Healthcare, Milwaukee, Wisconsin [n = 124]) or a 3.0T (Verio, Siemens, Erlangen, Germany [n = 23]; Discovery 750, GE Healthcare, Milwaukee, Wisconsin [n = 17]) MR scanner using an 8-channel or 32-channel head coil.

The imaging protocol included axial FLAIR, DWI, and ASL-PWI. ASL-PWI was performed using either a pseudocontinuous (n = 159) or pulsed (n = 5) labeling method. A quantitative perfusion map of CBF was obtained afterwards by fitting the signal intensity change between the labeled and control images to a previously published model [29]. Specific imaging parameters for all sequences are summarized in S1 Table.

### EEG acquisition protocol and analysis

EEG was recorded using the international 10–20 system and additional anterior temporal electrodes. EEG was acquired during or after seizures and analyzed by neurologists.

### Image analysis

#### Qualitative analysis

All images were visually analyzed for the presence or absence of the following: (1) diffusion restriction on DWI, (2) hyperintensity on fluid-attenuated inversion (FLAIR), and (3) perfusion abnormality on ASL-PWI. Image analysis was performed by two independent readers ([R. E. Y.] and [T. J. Y.] with 6 years and over than 10 years of experience, respectively), who were blinded to final diagnoses.

Perfusion abnormality on ASL-PWI, when present, was further analyzed in terms of the following: (1) pattern (hyper- or hypo- perfusion relative to the gray matter of normal contralateral parenchyma at the same slice), (2) multifocality (focal, multifocal, or hemispheric), and (3) atypical distribution against vascular territories (territorial if perfusion abnormality corresponded to one or more vascular territories, or non-territorial if not).

#### Quantitative analysis of CBF on ASL-PWI

For CBF quantification, one neuroradiologist (R. E. Y.) randomly placed six regions of interest of 10 mm^2^, three within the area of the perfusion abnormality (i.e., CBF_lesion_) and three within normal contralateral gray matter at the same level (i.e., CBF_gray matter_). Normalized CBF_lesion_ (nCBF_lesion_; nCBF_lesion_ = CBF_lesion_ / CBF_gray matter_) was derived to minimize possible biases due to interindividual variations in MR scanners and basal CBF.

### Statistical analysis

All statistical analyses were performed using a Statistical software program (MedCalc, version 11.1.1.0; MedCalc, Mariakerke, Belgium). For each parameter, normality of the data was assessed with the Kolmogorov-Smirnov test. Statistical significance was considered when *P* value was less than 0.05. Fisher exact test was used to compare incidences of various MR imaging findings between subgroups of seizures and between different final diagnoses. Sensitivity, specificity, and diagnostic accuracy for the differential diagnosis between seizures and seizure mimickers were calculated. nCBF values were compared using the Jonckheere-Terpstra trend test of independent samples with multiple pairwise comparisons. Interobserver agreement between two independent readers ([R. E. Y.] and [T. J. Y.]) regarding the presence of abnormal perfusion on ASL-PWI was assessed using linear κ coefficients [30].

## Results

### Demographics and final diagnoses of patients

Of the 164 patients included in this study, 89 were men [mean age, 57 years; age range, 19–91 years] and 75 were women [mean age, 55 years; age range, 23–81 years]. For seven patients who underwent MR imaging more than once due to recurrent episodes of seizure-like movements, only their initial MR images were included in the analysis.

Final diagnoses for the patients were as follows: 1) seizures (n = 129), 2) poststroke seizure (n = 18) (onset seizures [n = 12], early seizure [n = 0], late seizures [n = 6]), and 3) seizure mimickers (n = 17). The suspected etiologies of the seizure patients and time intervals between the last seizure events and MR scans are provided in the S1 Appendix. The seizure duration was unknown in 37 of 129 patients. Twenty-six of 92 patients with known seizure durations presented with status epilepticus. The median seizure duration for the other 66 patients were 3 minutes (interquartile range [IQR], 1–5). Presenting symptom was unknown in four of the 114 seizure cases with known time intervals past the last seizure events. Of the 110 cases with known symptoms, 75% (83 of 110) presented with convulsive symptoms, while 25% (27 of 110) presented with nonconvulsive symptoms. Meanwhile, 65% (71 of 110) were generalized seizures and 35% (39 of 110) were partial seizures.

### MR imaging findings

MR findings according to the final diagnoses are summarized in Table 1.

**Table 1:** Summary of MR imaging findings according to final diagnoses.

MR imaging findings	Seizure (n = 129)	Poststroke seizure (n = 18)			Seizure mimickers^c^ (n = 17)
		Onset seizure (n = 12)	Early seizure (n = 0)	Late seizure (n = 6)	
DWI diffusion restriction^a^	33 (26)	12 (100)	0 (0)	3 (50)	1 (6)^d^
FLAIR hyperintensity^a^	27 (21)	10 (83)	0 (0)	3 (50)	1 (6)^d^
ASL perfusion abnormality^a^	50 (39)	10 (83)	0 (0)	4 (67)	1 (6)^d^
Perfusion pattern					
Hyperperfusion	47 (94)	3 (30)^b^	0 (0)	3 (75)^b^	0 (0)
Hypoperfusion	3 (6)	7 (70)	0 (0)	1 (25)	1 (100)^d^
Multifocality					
Focal	27 (54)	4 (40)	0 (0)	4 (100)	0 (0)
Multifocal	15 (30)	5 (50)	0 (0)	0 (0)	1 (100)^d^
Hemispheric	8 (16)	1 (10)	0 (0)	0 (0)	0 (0)
Atypical distribution against vascular territories					
Territorial	1 (2)	5 (50)	0 (0)	3 (75)	1 (100)^d^
Non-territorial	49 (98)	5 (50)	0 (0)	1 (25)	0 (0)

Note: Unless otherwise specified, numbers in parentheses are percentages based on the number of patients with ASL perfusion abnormality.

ASL-PWI: arterial spin labeling perfusion-weighted imaging.

^a^ Numbers in parentheses are percentages based on the total number of patients.

^b^ Combined hypoperfusion was found in part of the infarcted area in all patients.

^c^ The ‘Seizure mimickers’ group included syncope (n = 8), psychogenic nonepileptic seizures (n = 4), orthostatic hypotension (n = 2), recurrent transient ischemic attack (n = 1), Bell’s palsy (n = 1), and acute ischemic stroke (n = 1).

^d^ Abnormal findings were revealed in one same patient with acute ischemic stroke.

#### Seizure group

Diffusion restriction on DWI and hyperintensity on FLAIR were present in 26% (33 of 129) and 21% (27 of 129) of the seizure patients, respectively. Perfusion abnormality on ASL-PWI was detected in 39% (50 of 129) of the seizure patients, most being the hyperperfusion pattern (94% [47 of 50]). Of the 50 patients with perfusion abnormality, multifocality or hemispheric involvement and atypical distribution against vascular territories were revealed in 46% and 98%, respectively. Both diffusion restriction on DWI and hyperintensity on FLAIR were present in 24 patients. Meanwhile, either diffusion restriction on DWI or hyperintensity on FLAIR was noted in 5 and 1 patient(s), respectively. Neither diffusion restriction on DWI nor hyperintensity on FLAIR was present in 20 patients (Table 1).

Perfusion patterns according to the time intervals between the last seizure events and MR scans and presenting symptoms are summarized in Tables 2 and 3.

**Table 2:** Perfusion patterns in seizure patients (convulsive vs. nonconvulsive) with known time intervals between the last seizure events and MR scans.

	Hyperperfusion	Hypoperfusion	Normal
Last seizure to MR scan			
≤ 5 hours (n = 38)	22 (58)	1 (3)	15 (39)
Convulsive (n = 25)	13 (52)	1 (4)	11 (44)
Nonconvulsive (n = 12)	8 (67)	0 (0)	4 (33)
Unknown (n = 1)	1 (100)	0 (0)	0 (0)
> 5 hours (n = 76)	20 (26)	1 (1)	55 (72)
Convulsive (n = 58)	13 (22)	1 (2)	44 (76)
Nonconvulsive (n = 15)	7 (47)	0 (0)	8 (53)
Unknown (n = 3)	0 (0)	0 (0)	3 (100)
Not identified (n = 15)			
*P* value^a^	.002	1	.001

Note: Numbers in parentheses are percentages.

^a^ *P* values were calculated by using Fisher exact test to determine whether the incidences of the perfusion pattern significantly differed between the patients in whom the time interval between the last seizure event and MR scan was ≤ 5 hours than in those in whom the time interval was > 5 hours.

**Table 3:** Perfusion patterns in seizure patients (generalized vs. partial) with known time intervals between the last seizure events and MR scans.

	Hyperperfusion	Hypoperfusion	Normal
Last seizure to MR scan			
≤ 5 hours (n = 38)	22 (58)	1 (3)	15 (39)
Generalized (n = 19)	10 (53)	1 (5)	8 (42)
Partial (n = 18)	11 (61)	0 (0)	7 (39)
Unknown (n = 1)	1 (100)	0 (0)	0 (0)
> 5 hours (n = 76)	20 (26)	1 (1)	55 (72)
Generalized (n = 52)	14 (27)	0 (0)	38 (73)
Partial (n = 21)	6 (29)	1 (5)	14 (67)
Unknown (n = 3)	0 (0)	0 (0)	3 (100)
Not identified (n = 15)			
*P* value^a^	.002	1	.001

Note: Numbers in parentheses are percentages.

^a^ *P* values were calculated by using Fisher exact test to determine whether the incidences of the perfusion pattern significantly differed between the patients in whom the time interval between the last seizure event and MR scan was ≤ 5 hours than in those in whom the time interval was > 5 hours.

ASL hyperperfusion was significantly more common in the patients in whom the time interval between the last seizure event and MR scan was ≤ 5 hours than in those in whom the time interval was > 5 hours (58% [22 of 38] vs. 26% [20 of 76], respectively; *P* = 0.002). Presenting symptom was unknown in four of the 114 seizure cases with known time intervals past the last seizure events. Of the 110 cases with known symptoms, 75% (83 of 110) presented with convulsive symptoms, while 25% (27 of 110) presented with nonconvulsive symptoms. Incidence of hyperperfusion did not significantly differ between the patients who presented with convulsive symptoms and those with nonconvulsive symptoms in both patients in whom the time interval between the last seizure event and MR scan was ≤ 5 hours (52% [13 of 25] vs. 67% [8 of 12], respectively; *P* = 0.491) and those in whom the time interval was > 5 hours (22% [13 of 58] vs. 47% [7 of 15], respectively; *P* = 0.100). Moreover, the incidences of hyperperfusion did not significantly differ between generalized seizures and partial seizures regardless of the time interval between the last seizure event and MR scan (53% [10 of 19] vs. 61% [11 of 18], respectively; *P* = 0.743 for ≤ 5 hour; 27% [14 of 52] vs. 29% [6 of 21], respectively; *P* = 1.000 for > 5 hours).

Data for follow-up MR imaging including ASL-PWI and DWI are provided in S2 Table.

#### Comparison of seizure and onset seizure in the poststroke seizure groups

Incidences of diffusion restriction on DWI, hyperintensity on FLAIR, and perfusion abnormality on ASL-PWI were significantly lower in seizures than in onset seizures within the poststroke seizure group (*P* < 0.001, *P* < 0.001, and *P* = 0.004, respectively). However, in the subgroup analysis, the incidence of hyperperfusion among the cases which showed ASL perfusion abnormality was significantly higher in seizures than in onset seizures (94% [47 of 50] vs. 30% [3 of 10], respectively; *P* < 0.001). Distinctively, the hyperperfusion in onset seizures within the poststroke seizure group showed combined hypoperfusion in part of the infarcted area in all patients. Therefore, the sole hyperperfusion without combined hypoperfusion was found exclusively in the seizure group. When the time interval between the last seizure and MR scan was ≤ 5 hours, the sensitivity, specificity, and diagnostic accuracy of the hyperperfusion pattern for the differential diagnosis between seizures and onset seizures were 58% (22 of 38), 75% (3 of 4), and 60% (25 of 42), respectively. Median nCBF_lesion_ in seizures was also significantly higher than that in onset seizures within the poststroke seizure group (2.236 [IQR, 1.820–2.807] vs. 0.472 [IQR, 0.309–1.954], respectively; *P* = .002). The Jonckheere-Terpstra trend test showed a statistically significant difference in the nCBF_lesion_ across the three different groups (i.e., generalized seizures, partial seizures, and onset seizures) (*P* = .027). In pair-wise comparisons, significant differences in the nCBF_lesion_ were observed between generalized seizures and onset seizures and between partial seizures and onset seizures (*P* < .05), but not between generalized seizures and partial seizures. In addition, the seizure group showed a significantly higher incidence of non-territorial distribution than onset seizures (98% [49 of 50] vs. 50% [5 of 10], respectively; *P* < 0.001) (Table 1).

#### Comparison of seizure and seizure mimicker groups

ASL perfusion abnormality was significantly more common in seizures than in seizure mimickers (*P* < 0.001) (Table 1). When the time interval between the last seizure and MR scan was ≤ 5 hours, the sensitivity, specificity and diagnostic accuracy of the hyperperfusion pattern for the differential diagnosis of seizures and seizure mimickers were 58% (22 of 38), 100% (17 of 17), and 69% (38 of 55), respectively (Tables 1 and 2).

#### Interobserver agreement for ASL-PWI findings

There was almost perfect interobserver agreement between the two readers regarding the presence of abnormal perfusion on ASL-PWI (*κ* = 0.873; 95% confidence interval, 0.796–0.949).

**Fig 1:**
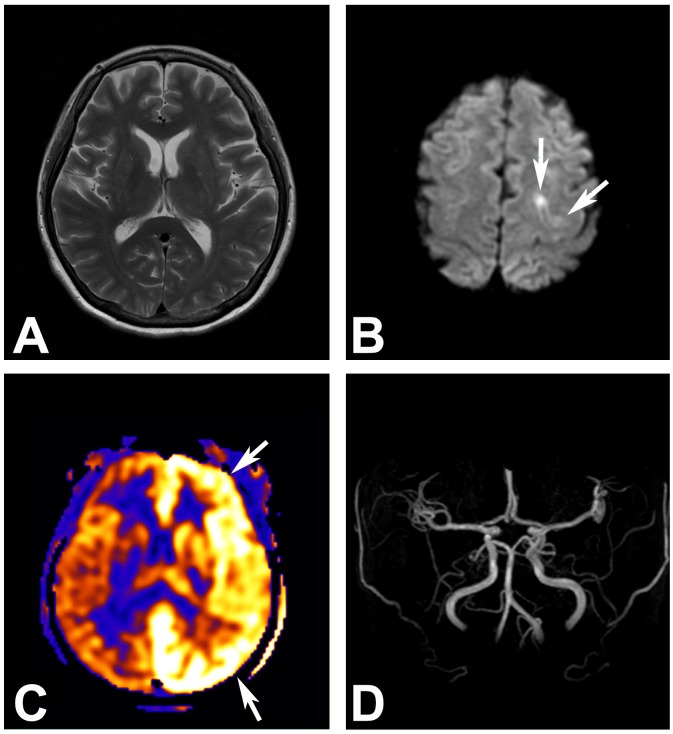
A 49-year-old woman who showed a convulsive seizure in the intensive care unit. (A) Brain parenchyma appears normal on the T2 FLAIR image. (B) Ill-defined and subtle diffusion restriction is noted at the left parietal cortex (arrows). (C) Arterial spin labeling perfusion-weighted image, however, clearly depicts hemispheric hyperperfusion (arrows) in the left cerebral hemisphere. (D) No apparent steno-occlusive lesion is found on the MR angiography.

**Fig 2:**
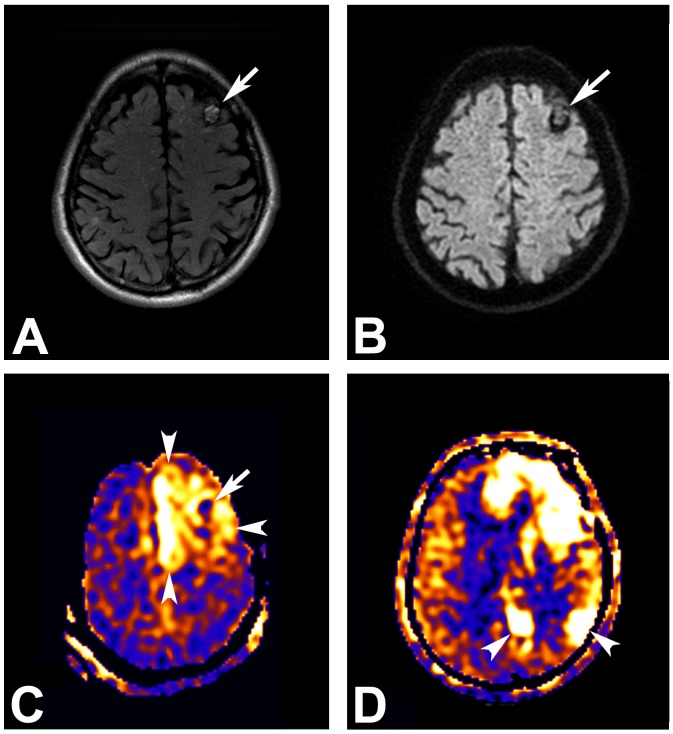
A 71-year-old man with underlying cavernous malformation who presented to the emergency department with a seizure. (A) T2 FLAIR MR image demonstrates a cavernous malformation (arrow) with a hemoderin rim and typical popcorn ball appearance secondary to multiple locules containing hemorrhage at the left frontal lobe. (B) Diffusion-weighted image shows no abnormal signal change around the cavernous malformation (arrow). (C) Arterial spin labeling perfusion-weighted MR image, however, reveals prominent perilesional hyperperfusion (arrowheads) surrounding the cavernous malformation (arrow) at the left frontal lobe. (D) Additional hyperperfusion foci (arrowheads) are also noted at the left parietal lobe.

**Fig 3:**
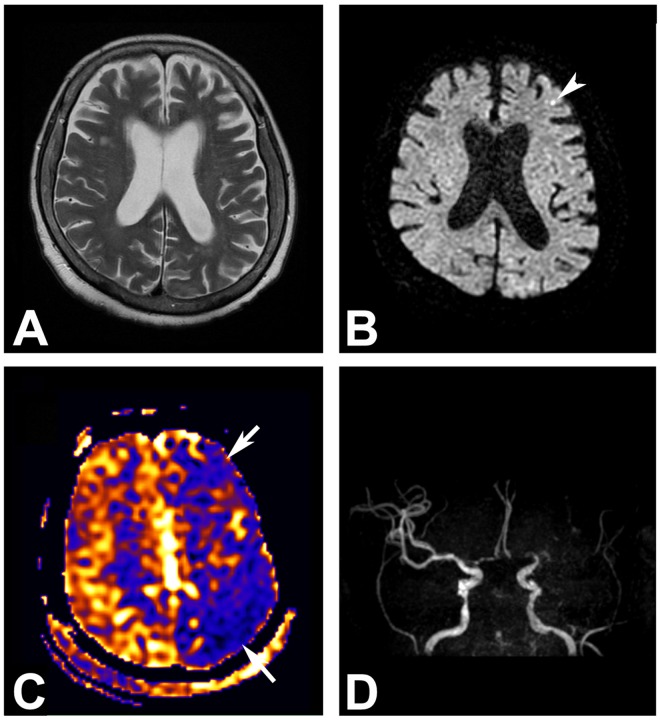
A 87-year-old woman who showed a convulsive seizure in the patient ward. (A) Brain parenchyma appears normal on the T2-weighted image. (B) A tiny dot-like diffusion restriction is noted at the left frontal cortex (arrowhead). Subtle cortical hyperintensities at the left occipital lobe are artifacts. (C) Arterial spin labeling perfusion-weighted MR image depicts hypoperfusion at the left middle cerebral artery territory (arrows). (D) MR angiography shows occlusion at the left M1 segment.

**Fig 4:**
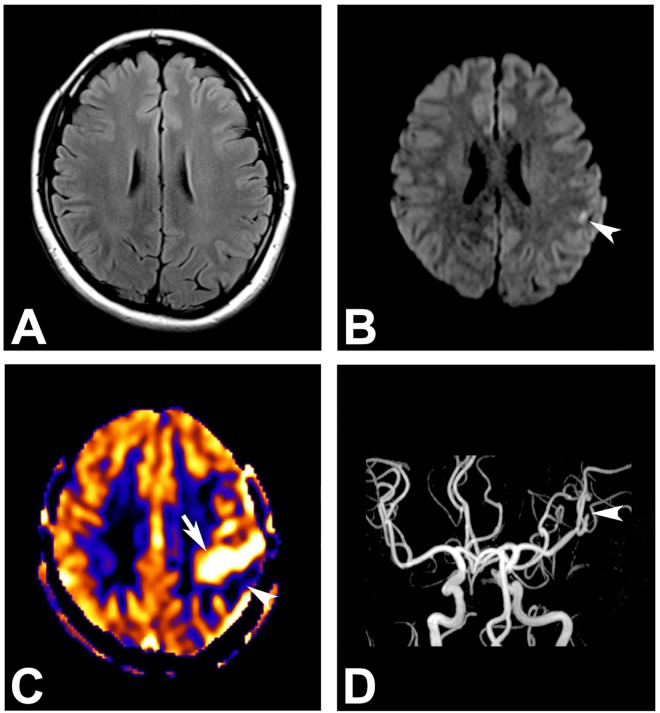
A 33-year-old woman who visited the emergency department for a nonconvulsive seizure. (A) Brain parenchyma appears normal on the T2 FLAIR image. (B) A tiny dot-like diffusion restriction is noted at the left parietal cortex (arrowhead). (C) Arterial spin labeling perfusion-weighted MR image depicts hyperperfusion at the left middle cerebral artery territory (arrow). Note the combined hypoperfusion portion adjacent to the hyperperfused area (arrowhead). (D) MR angiography reveals occlusion at the left M2 segment (arrowhead).

Representative MR images including ASL-PWI are shown in Figs 1, 2, 3 and 4.

### Correlation between EEG and ASL-PWI findings

Of the 164 cases, EEG results were available in 148 cases, including 120 seizures, 14 poststroke seizures, and 13 seizure mimickers. Among the 120 seizure patients, EEG and ASL-PWI localized seizure foci in 58 (48%) and 53 (44%) patients, respectively. In 31 patients, seizure focus could be localized on both EEG and ASL-PWI. With regard to the localization, EEG and ASL-PWI were concordant in 77% (24 of 31) and were discordant in 23% (7 of 31). Meanwhile, seizure focus was identified only on EEG in 27 patients and only on ASL-PWI in 22 patients. In 40 patients, neither EEG nor ASL-PWI revealed seizure foci. In the poststroke seizure group, seizure foci were localized on EEG and ASL-PWI in 36% (5 of 14) and 86% (12 of 14) of the cases, respectively. Both EEG and ASL-PWI identified seizure foci in four patients (concordant in four cases and discordant in 0 cases). Seizure focus could be identified only on EEG in one patient and only on ASL-PWI in eight patients. Both EEG and ASL-PWI failed to localize the seizure focus in one patient.

## Discussion

The results of the present study support that ASL-PWI can provide information about cerebral perfusion change in patients with seizures. Perfusion abnormality on ASL-PWI was detected in 39% of the seizure patients, most being the hyperperfusion pattern (94%). In addition, of the 50 patients with perfusion abnormality, multifocality or hemispheric involvement and atypical distribution against vascular territory were revealed in 46% and 98%, respectively. Particularly, hyperperfusion was more common in patients in whom the time interval between the last seizure and MR scan was ≤ 5 hours than in those in whom the time interval was > 5 hours.

A number of studies have documented the potential of ASL-PWI in depicting the perfusion abnormality in seizures. Pizzini et al. have demonstrated that 8 of 9 patients evaluated in the peri-ictal period showed hyperperfusion pattern, while the patients evaluated in the postictal period showed hypo- (7 of 10 patients) or normal (3 of 10 patients) perfusion pattern [10]. A few other studies have also shown hyperperfusion in the ictal or peri-ictal period and hypoperfusion in the postictal or interictal period [16, 17, 19, 21–27]. In keeping with the results, we found that the most common perfusion pattern observed in the peri-ictal period (defined according to the previous study by Pizzini et al. [10]) was hyperperfusion. Unlike the previous results, however, the most common pattern observed in the postictal period was normal pattern. Moreover, hyperperfusion pattern was also observed in some postictal scans with persistent hyperperfusion being noted in some patients even at follow-up (day 1–13). The possibility of the occurrence of unwitnessed seizures may explain the discrepancy between our results and those of the previous studies. On the other hand, Kanazawa et al. and Leonhardt et al. have also found persistent hyperperfusion in the postictal period, a finding in keeping with our results [9, 31]. The finding has been attributed to the possibility that the exact temporal resolution of ictal hyperperfusion could vary depending on the magnitude and duration of epileptic activities [31]. It has been also suggested that hyperperfusion may persist because of increased metabolism which attempts to restore the interictal state of neuronal excitability [9].

In addition, the incidence of hyperperfusion was found to be not significantly different between patients who presented with convulsive symptoms and those with nonconvulsive symptoms in the peri-ictal period, indicating that the cerebral perfusion is predominantly increased in the peri-ictal period regardless of the presenting symptom. The finding was consistent with the results of the previous study in which ictal ASL hyperperfusion along with cortical diffusion restriction facilitated the differential diagnosis of nonconvulsive partial status epilepticus from recurrent infarction in two patients, who presented with left hemiparesis and hemisensory disturbance [31].

With regard to the seizure semiology, hyperperfusion was the most common perfusion abnormality pattern in both generalized and partial seizures. In keeping with the fact that generalized seizures distort the electrical activities in bilateral hemispheres of the brain, some generalized seizures showed multifocality and bilateral involvement. Nonetheless, hyperperfusion involving the whole brain (bilateral hemispheres) was not seen in our patients and thus the hyperperfused area could be easily distinguished from the area with normal perfusion. Because the focally spared areas with normal perfusion was used for normalization in the quantitative analysis, nCBF_lesion_ was also increased in generalized seizures, thereby enabling discrimination of generalized seizures from onset seizures within the poststroke seizure group.

Another notable finding in the present study is that ASL-PWI can also aid in discriminating seizures from poststroke seizures. In between-group comparisons, the incidences of hyperperfusion and non-territorial distribution among the cases with ASL perfusion abnormality were significantly higher in seizures than in onset seizures within the poststroke seizure group. In clinical practice, acute ischemic stroke may mimic inhibitory (nonconvulsive) seizures or manifest as seizures. Onset seizures in the poststroke seizure group tended to show hypoperfusion as well as territorial distribution, despite the acquisition of ASL-PWI in the peri-ictal period. It has been suggested that early seizures after ischemic stroke may be caused by regional metabolic dysfunction and the release of excitotoxic neurotransmitters as a result of ischemic hypoxia [32]. Additionally, the previous study on PET findings of acute ischemic stroke patients has shown that ischemic penumbra may contain electrically irritable tissue with a potential to serve as a seizure focus [33]. Based on the previous findings, we speculate that hypoperfusion, instead of hyperperfusion, was predominantly noted in the onset seizures because ischemic penumbras were seizure foci in these patients. However, three cases (30%, 3 of 10) showed hyperperfusion. We assume that hyperperfusion in those settings might have resulted from either from recanalization or transient perfusion abnormality related to seizures, and a potential shortcoming of ASL-PWI is that it is often difficult to distinguish the two based on the perfusion imaging findings. However, according to the present data, the ASL-PWI finding of the sole hyperperfusion without combined hypoperfusion was found exclusively in the seizure group. Therefore, we speculate that the preferential perfusion abnormality pattern in seizures, specifically the sole hyperperfusion without combined partial hypoperfusion and non-territorial distribution, would be key imaging features for differentiating seizures from onset seizures in the poststroke seizure group.

In addition, ASL-PWI seems to have a potential to discriminate seizures from seizure mimickers. Several other nonepileptic disorders can also mimic seizures at clinical presentation, including syncope and psychogenic nonepileptic seizures. Unlike syncope for which thorough history taking plays a pivotal role in the diagnosis, distinguishing psychogenic nonepileptic seizures from epileptic seizures solely based on history taking or clinical presentation has been shown to be challenging in some cases [34]. In this study, we found that perfusion abnormality was absent in seizure mimickers except in acute ischemic stroke, suggesting that ASL-PWI would also be useful for differentiating seizures from seizure mimickers.

In the clinical setting, nuclear medicine perfusion techniques have been widely used for the evaluation of seizure activity [5, 35]. However, because these techniques cannot provide information about structural abnormality, acquisition of other structural imaging such as MR imaging is unavoidable. ASL-PWI, on the other hand, can be easily embedded in structural and functional MR protocols, and thus extensive information including structural abnormality, diffusion restriction, and/or functional abnormality as well as perfusion abnormality can be simultaneously obtained with ASL-PWI embedded MR imaging.

EEG is a widely used diagnostic tool for seizure patients in routine practice. Despite its common use in the seizure evaluation, EEG is shown to have a relatively low sensitivity ranging from 25 to 56% [36]. In this study, EEG failed to localize the seizure foci in 52% of the seizure patients. In those in whom seizure focus could be localized on both EEG and ASL-PWI, the concordance rate was 77%. Our result is in agreement with that of the previous report in which the concordance rate between ASL-PWI and clinical seizure foci, as determined by combined evaluation of seizure semiology, EEG, and conventional imaging modalities, was 74% [18].

Our study had a few limitations. First, the exact time intervals between last seizure events and MR scans and presenting symptoms could not be identified in some cases of suspected seizures with unwitnessed events. Second, for most seizure cases, we lacked follow-up ASL-PWI data to demonstrate the time course of perfusion abnormality. Third, the numbers of patients with final diagnoses other than seizures were small, presumably because the patients who met our inclusion criteria were those who underwent MR imaging for suspected seizures. Relatively large differences in the number of patients among different groups might have limited the statistical power for between-group comparisons. Fourth, CBF_lesion_ was normalized with respect to that of the normal contralateral gray matter to minimize possible biases due to interindividual variations in MR scanners and basal CBF, and the use of normalized CBF_lesion_ rather than absolute CBF values as in this study may hinder the detection of generalized hyperperfusion in generalized seizures. In this study, although some generalized seizures showed multifocality and bilateral involvement, hyperperfusion involving the whole brain (bilateral hemispheres) was not seen and thus the hyperperfused area could be easily distinguished from the area with normal perfusion, which was in turn used for CBF normalization. Therefore, nCBF_lesion_ was also increased in generalized seizures and was found to be significantly different between generalized seizures and onset seizures within the poststroke seizure group. Furthermore, unequivocally increased absolute CBF values exceeding the normal range may be another diagnostic clue for generalized hyperperfusion in case of bilateral hemispheric involvement in future studies. Fifth, a small proportion of the patients (5 of 164) were imaged using a pulsed ASL technique, which has been shown to have a relatively lower signal-to-noise ratio, as compared with a pseudocontinuous ASL technique.

In conclusion, our findings suggest that ASL-PWI can provide information regarding cerebral perfusion status in patients with seizures in acute settings. This technique may have the potential to be used as a noninvasive diagnostic tool to identify the cerebral perfusion in patients with seizures.

## Supporting information

S1 Table MR imaging parameters. (DOCX)

S2 Table Follow-up MR findings in seizure patients. (DOCX)

S1 Appendix Supplementary results. Suspected etiologies of the seizure patients and time intervals between the last seizure events and MR scans.
(DOCX)

## Abbreviations

ASL-PWI: arterial spin labeling perfusion-weighted imaging
CBF: cerebral blood flow
DSC-PWI: dynamic susceptibility contrast perfusion-weighted imaging
DWI: diffusion-weighted imaging
EEG: electroencephalogram
FLAIR: fluid-attenuated inversion recovery
nCBF: normalized cerebral blood flow
PET: positron emission tomography
SPECT: single-photon emission CT
