# Supplementary material for: Identification of cerebral perfusion using arterial spin labeling in patients with seizures in acute settings
Source: PLoS One. 2017 Mar 14;12(3):e0173538. doi: 10.1371/journal.pone.0173538 (PMC5349669; doi:10.1371/journal.pone.0173538)
Supplement: S1 Table — (DOCX) [file pone.0173538.s001.docx]

**S1 Table. MR imaging parameters.**

| Parameters | FLAIR | DWI | Pseudocontinuous  ASL-PWI^a^ | Pulsed  ASL-PWI^b^ |
| --- | --- | --- | --- | --- |
| Repetition time (msec) | 8000–8802 | 5500–10000 | 4446–4564 | 3000 |
| Echo time (msec) | 96–125 | 55–76 | 9.4–9.9 | 12 |
| Echo train length | 1–13 | 1 | 1 | 37 |
| Flip angle (degree) | 90–140 | 90 | 111–155 | 90 |
| Section thickness (mm) | 5 | 3–5 | 5–6 | 6 |
| Intersection gap (mm) | 1 | 1 | 0 | 1.5 |
| Field of view (mm) | 220 × 220 | 240 × 240 | 240 × 240 | 240 × 210 |
| Matrix | 320 × 192 | 160 × 160 | 128 × 128 | 64 × 49 |
| No. of signals acquired | 1 | 2 | 2–3 | 1 |
| No. of sections | 26 | 52–76 | 32–44 | 14 |

ASL-PWI: arterial spin labeling perfusion-weighted imaging. ^a^ The pseudocontinuous ASL-PWI was acquired at either a 1.5T (Signa HDxt, GE Healthcare, Milwaukee, Wisconsin [n = 124]) or 3.0T scanner (Discovery 750, GE Healthcare, Milwaukee, Wisconsin [n = 17]; Verio, Siemens, Erlangen, Germany [n = 18]). ASL-PWI = arterial spin labeling perfusion-weighted imaging. Labeling duration and postlabel delay were 1.5 sec and 1.5 sec, respectively. ^b^ The pulsed ASL-PWI was acquired at a 3.0T scanner (Verio, Siemens, Erlangen, Germany [n = 5]). Labeling duration and postlabel delay were 700 msec and 1100 msec, respectively.
