# Supplementary material for: Identification of cerebral perfusion using arterial spin labeling in patients with seizures in acute settings
Source: PLoS One. 2017 Mar 14;12(3):e0173538. doi: 10.1371/journal.pone.0173538 (PMC5349669; doi:10.1371/journal.pone.0173538)
Supplement: S2 Table — (DOCX) [file pone.0173538.s002.docx]

**S2 Table. Follow-up MR findings in seizure patients.**

|  | Initial | | F/U | | |
| --- | --- | --- | --- | --- | --- |
| Case | ASL  (Hyper- / hypoperfusion) | DWI^a^ | Day | ASL  (Hyper- / hypoperfusion) | DWI^a^ |
| 1 | Hyper- | + | 1 | Hyper- | – |
| 2 | Hyper- | + | 2 | Hyper- | – |
| 3 | Hyper- | – | 2 | Hyper- | – |
| 4 | Hyper- | + | 13 | Hyper- | + |
| 5 | Hyper- | – | 5 | Hypo- | – |

ASL-PWI: arterial spin labeling perfusion-weighted imaging. ^a^ + = present; – = absent.
