# Supplementary material for: Identification of cerebral perfusion using arterial spin labeling in patients with seizures in acute settings
Source: PLoS One. 2017 Mar 14;12(3):e0173538. doi: 10.1371/journal.pone.0173538 (PMC5349669; doi:10.1371/journal.pone.0173538)
Supplement: S1 Appendix — Suspected etiologies of the seizure patients and time intervals between the last seizure events and MR scans. (DOCX) [file pone.0173538.s003.docx]

**S1 Appendix**

**Supplementary Results**

Suspected etiologies of the seizure patients were as follows (in decreasing order of frequency): unknown (n = 30), metabolic cause (n = 22), intracranial hemorrhage (n = 13), postoperative cerebromalacia (n = 13), encephalitis (infectious and autoimmune) (n = 13), metastasis (n = 12), infarction (n = 5), hypoxic ischemia (n = 3), cavernous malformation (n = 3), treatment-related (chemotherapy or radiotherapy) (n = 3), posterior reversible encephalopathy syndrome (n = 3), calcification (n = 1), sepsis (n = 1), lymphoma (n = 1), moyamoya disease (n = 1), arteriovenous malformation (n = 1), mitochondrial encephalopathy with lactic acidosis and stroke-like episodes (n = 1), anaplastic astrocytoma (n = 1), Sturge-Weber syndrome (n = 1), and Rosai-Dorfman disease (n = 1).

Of the 129 seizure cases, the time past the last seizure event was unknown in 15 cases. Of the 114 seizure cases with known time intervals, 38 cases were scanned within 5 hours since the last seizure (range, 30 minutes to 5 hours) whereas 76 cases were scanned after 5 hours since the last seizure (range, 6 hours to 15 days). Regarding the onset seizures among poststroke seizures, time intervals between the last seizure events and MR scans were ≤ 5 hours (range, 1–4 hours) in four patients, > 5 hours (range, 6–26 hours) in four patients, and unspecified in four patients.
